# Spatial variability and influencing factors of topsoil organic carbon and total nitrogen in agricultural areas of North China

**DOI:** 10.3389/fpls.2026.1944206

**Published:** 2026-08-24

**Authors:** Jingyi Zhang, Yuanyuan Li, Qiong Yang, Peilin Su, Jie Chen, Zhenfu Wu

**Affiliations:** School of Agricultural and Biomanufacturing Zhengzhou University, Zhengzhou University, Zhengzhou, China

**Keywords:** agricultural areas North China Plain, digital soil mapping, random forest, soil organic carbon, soil total nitrogen

## Abstract

Soil organic carbon (SOC) and soil total nitrogen (STN) play crucial roles in the sustainability of soil production and ecological service function. Spatially explicit estimations of SOC and STN and the identification of factors influencing the spatial variability of them are prerequisites for formulating and implementing site-specific management measures to improve soil quality. Based on 613 topsoil observations and environmental covariates, this study revealed the spatial variability of SOC and STN in agricultural areas of North China employing the random forest (RF) model, and identified the controlling factors of them. The results indicated that SOC and STN in agricultural areas of North China displayed a similar spatial distribution pattern (high in the north and low in the south), with the mean value of 14.03 g kg^-1^ and 1.05 g kg^-1^, respectively. The spatial variability of SOC and STN in agricultural areas across of North China was found to be closely associated with the natural environmental factors such as mean annual temperature (MAT), mean annual elevation (MAE), elevation, et al. The spatial location, expressed in longitude, latitude and altitude coordinate values, was measured to explain the spatial variability of SOC and STN exceeding 40% and 30%, respectively, reflecting the value of spatial location as a covariate in digital soil mapping (DSM). The RF model using multi-temporal NDVI data had a higher accuracy and more stable output than using single-temporal data. Taking into account both model accuracy and simplicity, the multi-year monthly annual data (AvgMonths) of NDVI data was recommended to import RF in DSM. The results provide basic data for SOC and STN management tailored to local conditions in agricultural areas North of China, and also provide reference for optimizing the environmental covariate sets and their usage in DSM.

## 1 Introduction

Soil organic carbon (SOC) and soil total nitrogen (STN) serve as key indicators influencing soil productivity and regulating biogeochemical cycles. They are essential for the proper functioning of agroecosystems and the effective mitigation of global climate change (Bangroo et al., 2020; Zhang et al., 2020). The spatial prediction of SOC and STN on farmland, along with uncovering the natural and human factors influencing their spatial variability, holds immense importance (Ma et al., 2022). It contributes significantly to understanding the C-N cycle mechanism in agroecosystems and maintaining carbon and nitrogen balance at the field, landscape, and regional scales (Komolafe et al., 2021). It is a prerequisite for implementing the “double carbon” strategy, preventing and controlling agricultural carbon non-point source pollution, promoting agricultural sustainable development and ecological civilization construction (Liao et al., 2026; Baveye et al., 2018).

The spatial variability of SOC and STN across various scales has been extensively researched and analyzed by domestic and foreign scholars. The findings indicate that SOC and STN exhibit non-uniformity and spatial variability, forming a dynamic continuum (Zhang et al., 2025; Batjes, 2014). The content of SOC and STN is influenced by a range of driving factors. These factors are intricately linked to natural elements like climate, topography, biology, parent material, and soil physical and chemical properties (Abebe et al., 2020). Additionally, human agronomic activities, including tillage, fertilization, irrigation, crop rotation, straw and stubble management, play a significant role in this dynamic interaction (Arunrat et al., 2022). The heterogeneity of these factors in both time and space leads to the spatio-temporal variability of SOC and STN levels (Al Shoumik and Khan, 2023). As a result, the distinct spatial variability characteristics arise from the influence of various natural and human factors (de Anta et al., 2020; Kunkel et al., 2019). In earlier studies focusing on soil spatial variability, scholars typically examined small, regionally representative agricultural areas (Tian et al., 2023). The majority of these studies encompassed county, city, and provincial regions (Jiang et al., 2024; Cao et al., 2016; Hu et al., 2014). Soil spatial variability is scale-dependent, and the autocorrelation of the same variable varies significantly across different scales (Li et al., 2022; Ma et al., 2021). Research scale determines the dominant factors controlling soil property variability: studies conducted across larger regions tend to capture broad-scale spatial patterns primarily governed by climatic and topographic factors, whereas studies with finer sampling intervals at smaller extents are more likely to reveal local-scale heterogeneity shaped by anthropogenic activities (Kebonye et al., 2024; Goenster-Jordan et al., 2018). On county and city scales, the primary influencing factors on soil properties were the amounts of nitrogen fertilizer application and straw return (Abebe et al., 2020; Li et al., 2023). Contrastingly, at provincial and higher scales, natural environmental factors like mean annual precipitation and mean annual temperature played a more prominent role in influencing soil properties (Zhou et al., 2023). The general rule states that at smaller scales, human factors have a more pronounced relative effect on the spatial differentiation of soil properties. Conversely, at larger scales, natural factors like climate and topography exert a greater relative influence on the spatial differentiation of soil properties.

DSM has emerged as the primary method for studying the spatial variability prediction of soil properties (Minasny and McBratney, 2016). It involves organizing and establishing a spatial soil information system through field observation and laboratory analysis, integrating both spatial and non-spatial components in the soil prediction system (Veloso et al., 2026; Lagacherie and McBratney, 2006). Given the establishment of soil sample points, the output results are predominantly influenced by environmental covariates, sampling strategies, prediction models, and the specific attributes of the target soil (Zhang et al., 2023). The core technical framework of DSM is the SCORPAN model proposed by McBratney et al. based on Dokuchaev’s soil genetic theory and Jenny’s soil state factor equation (McBratney et al., 2003). The relationship between soil and environment is nonlinear. Machine learning (ML) algorithm, as one of the most important attribute space prediction models, can effectively integrate, process and utilize multi-source data of different types and resolutions without making any statistical assumptions on the data before model fitting. It is well-suited for fitting and uncovering the intricate nonlinear relationship between target soil types and properties, along with various covariates like other soil factors and environmental elements (Heung et al., 2016; Yang et al., 2021). In the realm of machine learning, the random forest (RF) model stands out as one of the most widely adopted algorithms, and has been shown to perform well in predicting soil properties compared to many other machine learning methods. This is attributed to its advantages, including high prediction accuracy, reduced overfitting, and robust noise tolerance (Hengl et al., 2018; Makungwe et al., 2021).

As rapid access to soil data and environmental information, particularly remote sensing data, continues to advance, and shared computing power improves, numerous scholars, building upon the SCORPAN model, have sought to broaden the covariate set (Gardin et al., 2021). This expansion extends beyond conventional factors (climate, organisms, terrain, parent material, soil age, and spatial location) to encompass remote sensing data and human management practices. Nevertheless, the quest for predictive covariates is ongoing, with many studies still in exploration (Zeraatpisheh et al., 2022). Remote sensing variables, thanks to their extensive coverage, easy acquisition, and the cost-effectiveness of satellite image data, have evolved into a data source with vast application prospects. They actively contribute to the spatial variability prediction of soil attributes, including SOC and STN (Grinand et al., 2017; Li et al., 2021). The predictive mapping performance of DSM is intricately tied to the spatial structural characteristics of the target soil properties (Pouladi et al., 2023; Tian et al., 2022). In the SCORPAN model, spatial location is considered one of the constitutive models. In the current spatial prediction of SOC and STN, particularly in regions beyond the provincial level, there is limited research on the influence of spatial position in the SCORPAN model.

The objective of this study is to reveal the spatial variability of topsoil organic carbon and total nitrogen in agricultural areas of North China based on the output of DSM, to identify the controlling factors of them, and to explore the impact of utilization of multi-temporal remote sensing data and spatial location data on DSM results.

## 2 Materials

### 2.1 Study area

**Figure 1:**
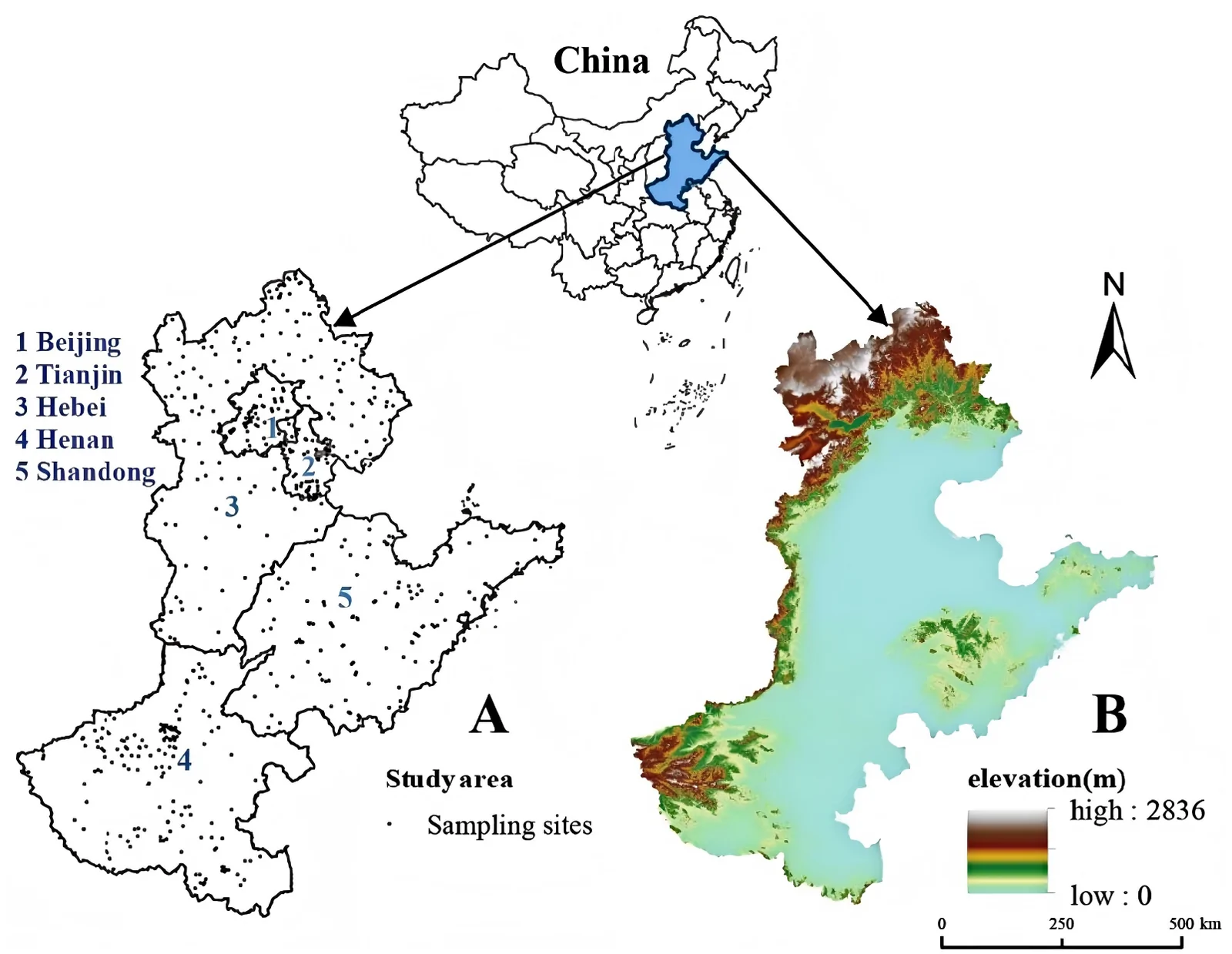
Geographical location of the study area, spatial variability of soil sample sites **(A)** and digital elevation model **(B)**.

The North China Agricultural Region refers to the intensive agricultural area comprising five northern provinces and municipalities, namely Beijing, Tianjin, Hebei, Henan, and Shandong. Situated in northern China within the North China Plain, with a total area of 5.40×10^5^ km^2^ (31°23’ -42°24’ N and 110°21’ -122°42’ E). The terrain is characterized by significant undulations and comprises mountains, hills, plains, and basins. Overall, it exhibits a west-to-east trend of higher elevations in the west and lower elevations in the east, ranging from 0 to 2855 m altitude (**Figure 1B**). The study area is situated in the temperate and warm temperate zones, characterized by a temperate monsoon climate. The northern part is closer to the temperate continental climate, while the southern part is closer to the subtropical monsoon climate. The mean annual temperature ranges from -1.0 to 16.6 °C, with a mean annual precipitation of 327.3-1433.5 mm, making it highly conducive to crop growth. By 2022, the total cultivated land area in the study area is projected to reach 204.33 million km2, representing 16% of China’s total cultivated land area. The study area achieved a total grain output of 1,615.7 billion kg, constituting 24% of the nation’s total grain output. Throughout most regions in the study area, a cultivated land rotation system involving wheat or rice, corn or rape, and corn and soybean was implemented. Based on the Chinese Soil Taxonomy (3rd edition), the major agricultural soils in the study area encompass various orders, including Anthrosols, Halosols, Gleyosols, Isohumosols, Argosols, Cambosols, Primosols.

### 2.2 Soil samples

The soil sample data were collected during the national land survey conducted between 2009 and 2013. The survey points for cultivated land soil profiles in the study area were sourced from Soil Series of China, specifically the volumes for Beijing-Tianjin, Hebei, Henan and Shandong. Data on surface STN, SOC, soil attribute covariates, and coordinates for each point were entered into Excel. The plots were conducted using the ArcGIS 10.7 software. The geographical coordinate system selected was World Geodetic System 1984 (WGS84), which was then transformed into WGS_1984_UTM_Zone_49N as the projection coordinate system. A total of 613 sample points were obtained (**Figure 1A**). The sample collection and processing in this study adhered strictly to the guiding principles outlined in the agricultural industry standard NY/T1121 of the People’s Republic of China. Each sampling point is randomly chosen, and soil samples of the same depth are selected in equal proportions. Multiple sampling points from a sampling unit are combined to create samples for the sampling point, enhancing the representativeness of the samples. The sample points and basic data in this paper encompassed SOC and STN data from the provinces of Beijing, Tianjin, Hebei, Henan, and Shandong. The collected samples underwent air-drying and were screened through a 2 mm screen. The SOC method employed potassium dichromate sulfuric acid digestion, while the STN method involved potassium dichromate sulfuric acid digestion-distillation. The dataset comprised 604 SOC samples and 464 STN samples.

### 2.3 Covariate pool

Based on the SCORPAN spatial prediction function quantified by McBratney et al., the covariates employed to predict target soil variables should encompass other soil properties and factors, including climate, organisms, terrain, parent material, soil age, and spatial location. In this study, leveraging SCORPAN, we utilized 51 environmental covariates across six aspects: spatial location, climate, terrain, organisms, management practices, and soil properties. These covariates exhibit correlation or cooperative spatial changes with SOC and STN. The pertinent information of environmental covariates is presented in the following six aspects:

Spatial location data encompass x-coordinate, y-coordinate, and elevation after projection. The primary source for these data is the x-coordinate and y-coordinate information extracted from Soil Series of China volumes for Beijing-Tianjin, Hebei, Henan and Shandong. The elevation data was obtained from the ALOS phased Array L-band Synthetic Aperture Radar (PALSAR) with a resolution of 12.5m. From NASA’s official website (https://search.asf.alaska.edu/#/).

Climate data encompasses mean annual precipitation, mean annual temperature, mean annual evaporation, atmospheric nitrogen dry deposition, atmospheric nitrogen wet deposition, and mean annual relative humidity. The raster data for mean annual precipitation, mean annual temperature, and mean annual evaporation are obtained from Resource and Environmental Science Data Platform (https://www.resdc.cn/). Raster data for atmospheric nitrogen dry deposition and atmospheric nitrogen wet deposition are sourced from National Science & Technology Infrastructure (http://rs.cern.ac.cn/index.jsp).

Terrain data encompass geomorphology, aspect, slope, total curvature, planar curvature and profile curvature. Geomorphology is extracted from the Spatial variability data for 1:1 million geomorphic types in China. Other variables derived from DEM are computed using 12.5-meter resolution DEM data with ArcGIS 10.7 software.

Organisms data encompass the net primary productivity (NPP) and the normalized difference vegetation index (NDVI) based on four years and 48 months of data from 2010 to 2013, respectively. The NDVI data is sourced from the Vegetation Index product of the MODIS satellite (MOD13Q1) with a spatial resolution of 250 meters and a temporal resolution of 16 days. NPP data is obtained from China’s Resources and Environment Science Data Center (http://www.resdc.cn/Default.aspx), providing a synthesis of 8 MODIS satellite images.

Management practice data encompass land use and land cover change (LUCC), amounts of fertilizer applications, and amounts of straw return. LUCC data is sourced from the Global Soil Cover Product 2020 produced by ESA, based on Sentinel-1 and Sentinel-2 data, with a spatial resolution of 10 meters. Amounts of fertilizer applications and amounts of straw return to the field are derived from the 2013 Statistical Yearbook of Beijing, Tianjin, Hebei, Henan, and Shandong. The amount of straw returned to the field was calculated based on the yield of main crops.

Soil properties data encompass soil types, soil sand content, soil silt content, soil clay content, pH value, cation exchange capacity, soil inorganic carbon, soil available phosphorous, and soil available potassium. Soil properties data are extracted from sample data in Soil Series of China volumes for Beijing-Tianjin, Hebei, Henan and Shandong. Raster data is produced through ordinary kriging interpolation using ArcGIS 10.7 software.

**Table 1** displays concise information about the covariates.

**Table 1:** List of covariates and resolution used in the study.

Categories	Covariates	Abbreviation	Resolution
Spatial location	Projected coordinates x	Pointx	Samples^1^
	Projected coordinates y	Pointy	Samples^1^
	Elevation	Elevation	12.5m
Climate	Mean Annual Precipitation	MAP	1km
	Mean Annual Temperature	MAT	1km
	Mean Annual Evaporation	MAE	1km
	Atmospheric Nitrogen Dry Deposition	ANDD	10km
	Atmospheric Nitrogen Wet Deposition	ANWD	1km
	Mean Annual relative Humidity	MAH	1km
Terrain	Geomorphology	Geomor	1km
	Aspect	Aspect	12.5m
	Slope	Slope	12.5m
	Total Curvature	TotalC	12.5m
	Planar Curvature	PlanarC	12.5m
	Profile Curvature	ProfileC	12.5m
Organisms	Net Primary Productivity	Npp	1km
	Normalized Difference Vegetation Index	Ndvi	1km
Management practices	Land use and Land cover change	LUCC	10m
	Amounts of Fertilizer Applications	FAA	Municipal level^2^
	Amounts of Straw Return	SRA	Municipal level^2^
Soil properties	Soil types	Soiltypes	1km
	Soil sand content	Sand	1km
	Soil silt content	Silt	1km
	Soil clay content	Clay	1km
	pH value	pH	1km
	Cation exchange capacity	CEC	1km
	Soil inorganic carbon	SIC	1km
	Soil available phosphorous	SAP	1km
	Soil available potassium	SAK	1km

^1^ “Samples” refers to the central coordinates of the 1 km × 1 km prediction units.

^2^ “Administrative unit” indicates that the data were derived from statistical yearbooks at the municipal or provincial level.

### 2.4 Variable selection and importance measurement

In many machine learning, the use of environmental covariates is prone to redundancy and may have negative effects, leading to a decrease in prediction accuracy. Therefore, prior to model establishment, it is essential to preprocess the environmental covariates of the target, involving variable selection or feature selection. In this study, the Boruta feature selection algorithm is employed (Keskin et al., 2019). This algorithm randomly samples and selects each variable involved in modeling, combines the obtained original and shadow variables, trains the RF model, calculates the importance score of each variable (Z-score), and subsequently ranks the variables based on their importance scores. For subsequent modeling, only the original features that surpass the importance of the shadowmax feature are utilized. This study applied the Boruta algorithm using the Boruta package (Kursa et al., 2010) in R 4.2.1.

The Boruta algorithm employs the random forest model to compute the Z-score for each variable by utilizing both raw variables and shadow variables. The importance score is influenced by the size of the original data. To ensure comparability with the Boruta output tables of SOC and STN, it is crucial to employ standardized importance score comparisons. In this study, the following standardized importance scores were utilized as relative importance indices:


$${\text{Z-score}}_{stand}=\frac{Z\text{-score}}{Z{\text{-score}}_{shadowmax}}-1$$

Where Z-scorestand represents the standardized Z-score, signifying the standardized importance score employed in this study; Z-score corresponds to the original importance score generated by the Boruta algorithm; Z-score_shadowmax_ denotes the maximum shadow feature. Thus, when Z-score_stand_ > 0, it indicates that the variable is an important factor, and the greater the score, the more significant it is.

### 2.5 Spatial prediction of topsoil organic carbon and total nitrogen

The relationship between soil properties and environmental covariates is intricate and nonlinear. Traditional statistics such as linear regression fall short of meeting the requirements of DSM. An increasing number of studies are opting for machine learning approaches, with Random Forest (RF) being a popular choice (Breiman, 2001). RF is an integrated learning algorithm based on a tree structure, adept at handling both classification and regression problems, and it serves as an extension of the CART model. In this study, employing optimization screening, the ntree parameter is set to 1000, and the mtry parameter is set to 3, revealing that the RF model attains the lowest error rate under these conditions. The randomForest package is utilized for programming in the R 4.3.1 environment to construct the prediction model.

This study assessed the difference in DSM performance for SOC and STN soil attributes through 10-fold cross-validation. When evaluating model performance, the commonly used metrics include the coefficient of determination (R^2^) and root mean square error (RMSE), enabling a comparison of the accuracy of spatial prediction for soil properties across different sets of environmental covariates.


$$R^{2}={\left(\frac{\sum_{i=1}^{n}\left(O_{i}-\bar{O}\right)\left(M_{i}-M\right)}{\sqrt{\sum_{i=1}^{n}{\left(O_{i}-\bar{O}\right)}^{2}\sqrt{\sum_{i=1}^{n}{\left(M_{i}-M\right)}^{2}}}}\right)}^{2}$$

$$RMSE=\sqrt{\frac{\sum_{i=1}^{n}{\left(O_{i}-M_{i}\right)}^{2}}{n}}$$

Where O_i_ is the measured value, M_i_ is the predicted value, $\bar{\text{O}}$ and M are the means of the predicted and measured values, respectively, and n is the sample point number. It is assumed that the predicted values of the Random Forest (RF) model based on bootstrap iteration follow a normal distribution. Through 10 bootstrap iterations, effective estimation of the predicted normal distribution parameters (mean μ and standard deviation σ) for SOC and STN is achieved. The 10 standard deviations of prediction results, obtained from uncertainty calculations related to RF predictions, contribute to quantifying the uncertainty of SOC and STN through probability variability. Utilizing their mean (μ) and standard deviation (σ), a 90% confidence interval (CI) derived interval plot for SOC and STN is obtained using the formula μ ± 1.645σ. Ultimately, the lower limit (5th percentile), average, and upper limit (95th percentile) of SOC and STN observations are leveraged to quantitatively predict soil properties and their uncertainties. This approach aligns with the GlobalSoilMap technical specifications (Arrouays et al., 2014).

## 3 Results

### 3.1 Statistical analysis of topsoil organic carbon and total nitrogen observations

**Table 2** displays the descriptive statistics for topsoil organic carbon and total nitrogen content data from sample points in the study area. The samples comprise 604 organic carbon data points (SOCsample) with a variation range of 0.54~58.20 g kg^-1^ with μ = 14.03 g kg^-1^, σ = 10.45 g kg^-1^, and the coefficient of variation is 74.49%. Additionally, there are 464 STNsamples with total nitrogen data, ranging from 0.08 to 3.45 g kg^-1^ with μ = 1.05 g kg^-1^, σ = 0.61 g kg^-1^, and the coefficient of variation is 58.21%. Both datasets exhibit a moderate degree of variation.

**Table 2:** Descriptive statistics of topsoil organic carbon and total nitrogen observations in the study area.

Soil properties	n	Mean(g kg^-1^)	Max(g kg^-1^)	Min(g kg^-1^)	SD(g kg^-1^)	CV
SOC	604	14.03	58.20	0.54	10.45	74.49
STN	464	1.05	3.45	0.08	0.61	58.21

SD, standard deviation; CV, coefficient of variation.

**Figure 2:**
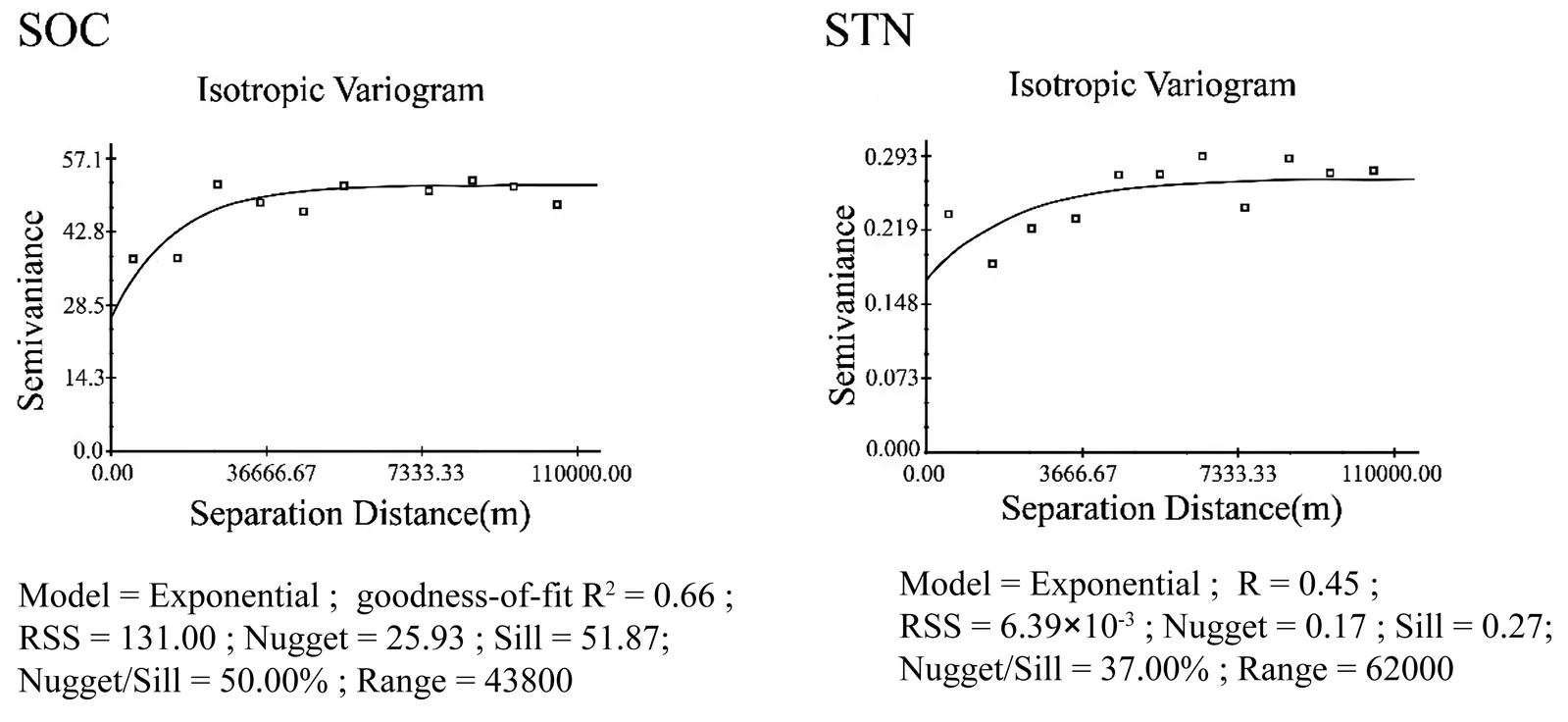
Optimal semi-variance model of topsoil organic carbon and total nitrogen.

To assess the spatial variability of SOC and STN, a semi-variance model (**Figure 2**) for surface SOC and STN was proposed. The best-fitting model for surface SOC is an exponential model with an R^2^ value of 0.66, a nugget value of 25.93, and a sill value of 51.87. The nugget effect is 50.00%. The optimal model for surface STN is also an exponential model with an R^2^ value of 0.45, a nugget value of 0.17, and a sill value of 0.27. The nugget effect is 37.00%. The nugget effect for SOC and STN indicate a strong spatial dependence for both variables.

### 3.2 Relative importance of environmental covariates

**Figure 3:**
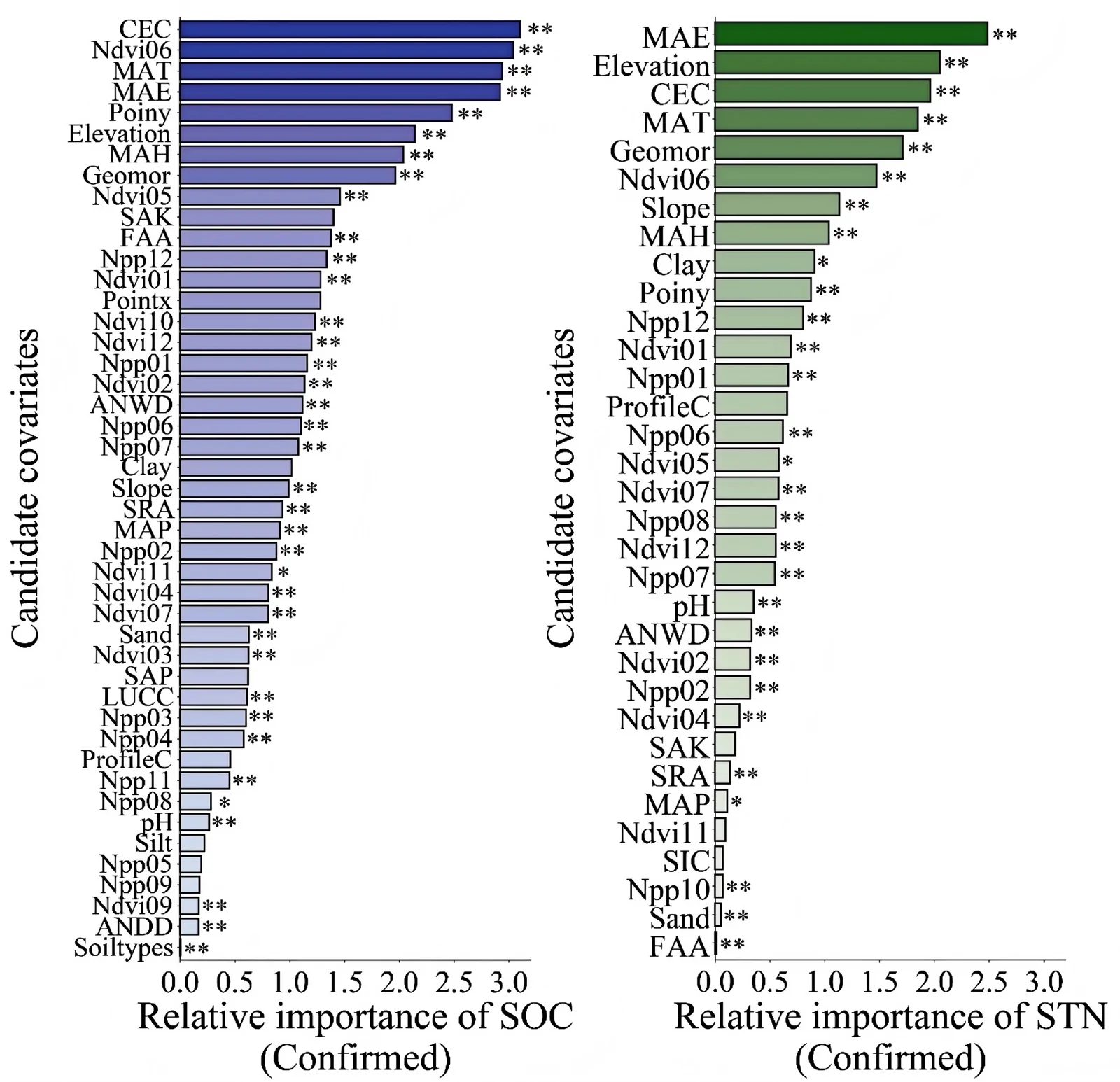
Relative importance of covariates for topsoil organic carbon and total nitrogen prediction calculated using the Boruta algorithm (Only the confirmed covariates are shown). * and ** indicate the significant levels of Pearson’s correlation coefficient between continuous covariates and target variable at p< 0.05 and p< 0.01, respectively.

Selecting the appropriate environmental covariates is crucial for improving the prediction performance of RF model. We chose 51 environmental covariates, including 12 NDVI and 12 NPP remote sensing variables. This study employed the Boruta algorithm and was relatively standardized according to section 2.4. The results are presented in **Figure 3** SOC includes 45 important variables, and STN includes 33 important variables. In predicting topsoil organic carbon content, the relative importance of cation exchange capacity, June NDVI data, mean annual temperature, and mean annual precipitation reached 2.5. Projected coordinates y, elevation, and mean annual relative humidity reached 2.0. In the prediction of topsoil total nitrogen content, no environmental covariate reached 2.5, only mean annual precipitation and elevation reached 2.0. The covariate set selected in this study is more conducive to the spatial prediction of soil organic carbon content.

### 3.3 Model predictive performance and uncertainty analysis

Using the confirmed environmental covariates in section 3.2 as input variables for the RF model, we constructed the model to explore the spatial differentiation characteristics of SOC and STN. According to the accuracy evaluation of model performance in section 2.5, relevant results are shown in **Table 3**. The performance indices indicate that the selection of an appropriate environmental covariate set has a positive impact on the prediction performance of SOC and STN content.

**Table 3:** Prediction performance of topsoil organic carbon and total nitrogen content (R^2^and RMSE) in the study area.

Soil properties	R^2^	RMSE
SOC	0.53	7.39
STN	0.44	0.45

**Figure 4:**
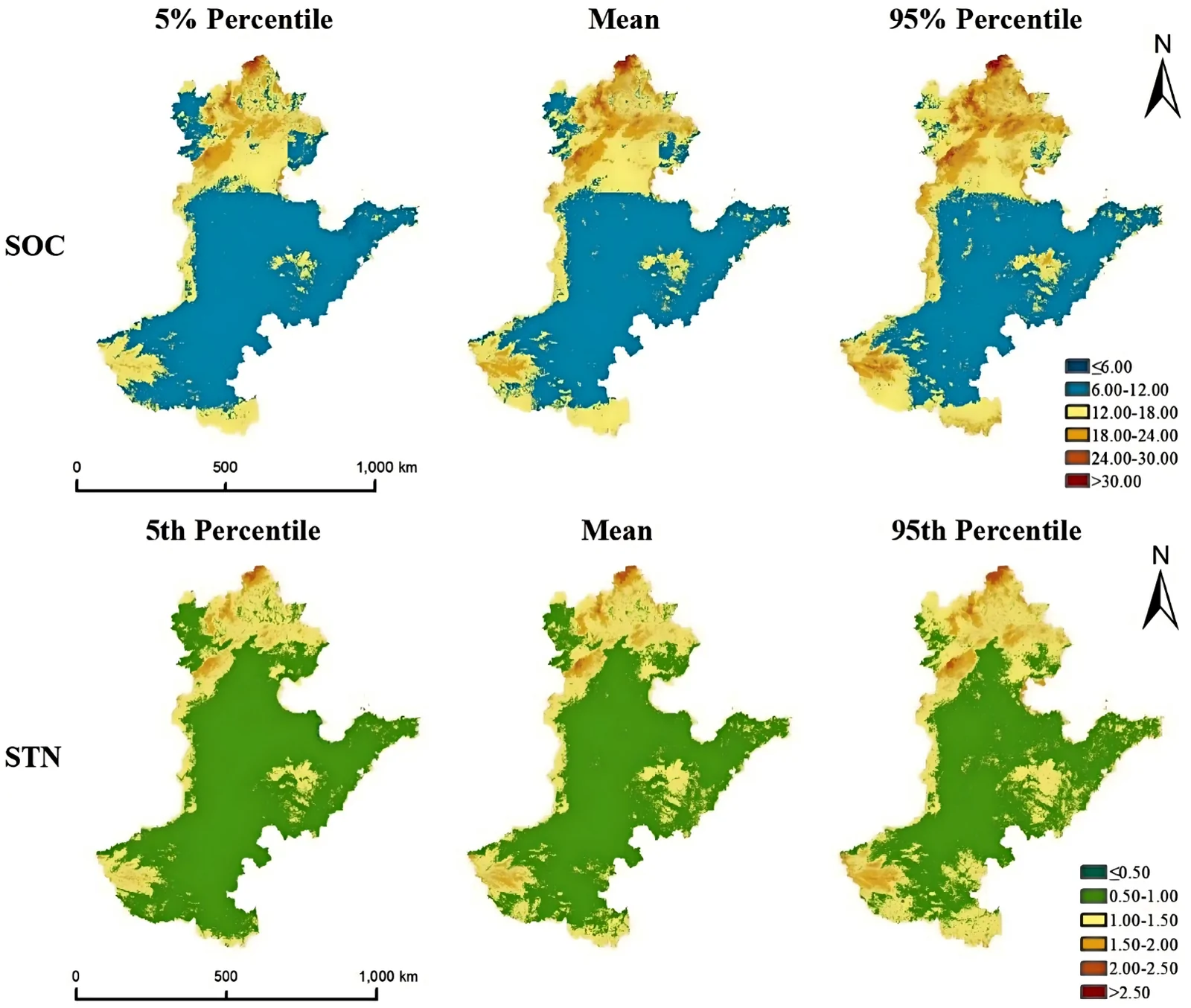
Prediction of lower (5% percentile), average (mean), and upper (95% percentile) limits for topsoil organic carbon and total nitrogen using the Random Forest model.

Based on the uncertainty analysis in section 2.5, **Figure 4** illustrates the lower limit (5th percentile), average value, and upper limit (95th percentile) of SOC and STN predictions using the random forest model. From spatial location, SOC and STN in this study area exhibit similar spatial variability characteristics, displaying a pattern of “high in the north and low in the south.” This variability aligns with the geomorphic type map of the study area, where mountains and hills typically have higher SOC and STN content. The overall distribution pattern of SOC in North China closely resembled the national-scale prediction of organic matter in the 1980s (Liang et al., 2019).

## 4 Discussion

### 4.1 Effect of multi-temporal NDVI on SOC and STN spatial prediction accuracy

Remote sensing data, with its wide coverage, convenient acquisition, and low cost of satellite image data, has gradually become a data source with broad application prospects (Dharumarajan et al., 2017; Li et al., 2021). It is actively involved in predicting various soil properties in DSM. Typically, remote sensing cannot directly acquire soil information due to vegetation coverage. However, as vegetation influences soil biological processes, it plays a role in the spatial variability of soil properties (Ballabio et al., 2012; Li et al., 2019). In previous studies, the use of remote sensing data, especially multi-temporal remote sensing data, to participate in DSM can effectively improve the prediction ability of the model (He et al., 2021; Zhou et al., 2020). Previous studies have demonstrated that incorporating remote sensing data, particularly multi-temporal remote sensing data, into DSM can effectively enhance the predictive capability of the model. Nevertheless, the utilization of the remote sensing variable NDVI is seldom examined in this context. This study explores two directions, namely, monthly data for a single year and the monthly average data spanning several years. The monthly data for the single year consists of the 12 months from 2010 to 2013, with each month serving as a predicted environmental covariate. The multi-year monthly average data is derived from the four-year data spanning 2010 to 2013. The ArcGIS 10.7 software is employed to calculate the average of raster data, resulting in the generation of 12-month monthly average NDVI data, denoted as AvgMonths, which serves as an environmental covariate.

**Figure 5:**
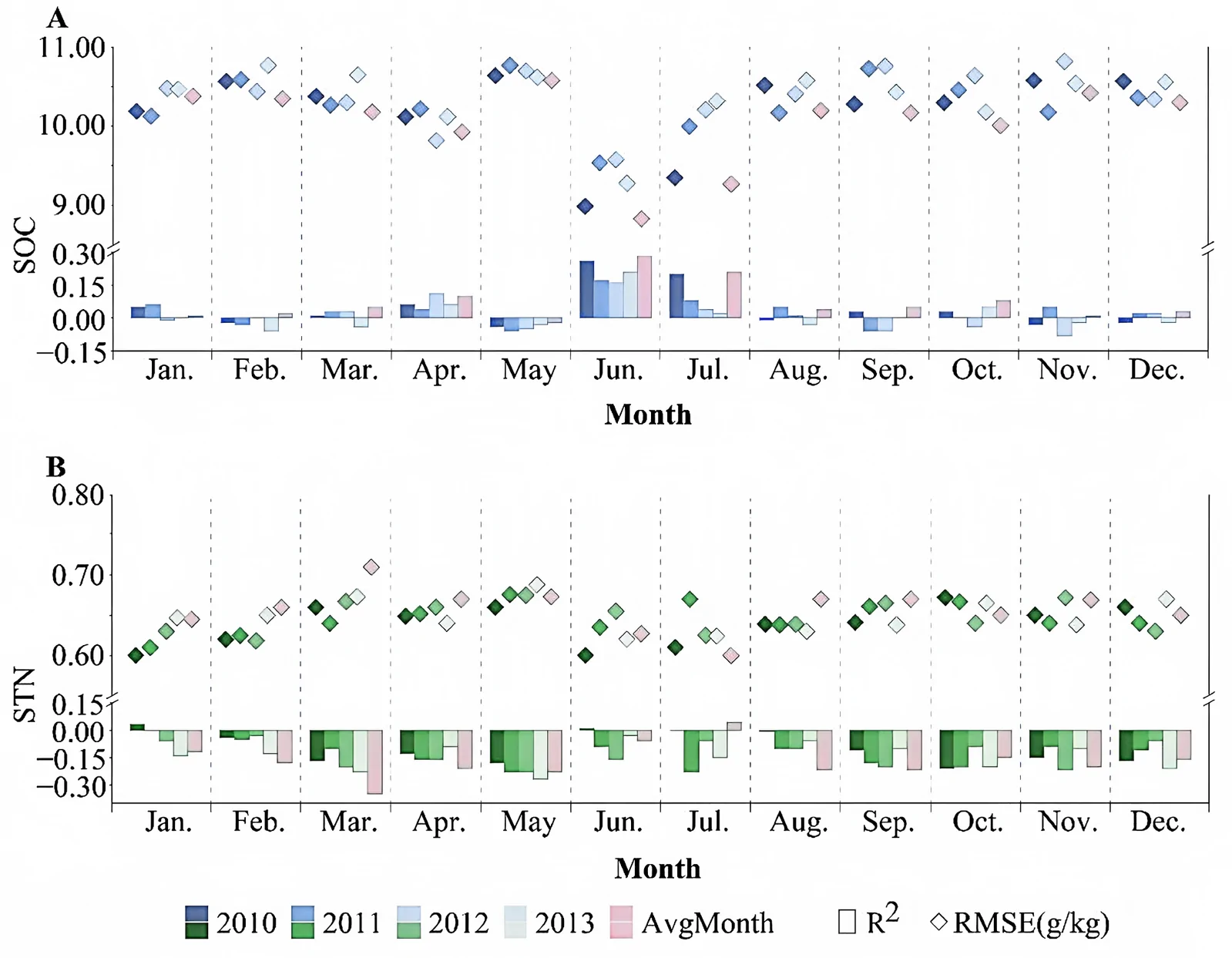
Single-temporal remote sensing data was used to predict the performance of **(A)** topsoil organic carbon (SOC) and **(B)** topsoil total nitrogen (STN).

Illustrated in **Figures 5A, B**, single-temporal NDVI data were employed as environmental covariates for predicting SOC and STN in the study area. The prediction model’s R^2^ and RMSE were subsequently determined. The results indicate that the use of single-temporal NDVI data as environmental covariates leads to substantial fluctuations in R^2^ and RMSE, resulting in an unstable prediction performance (Zhao et al., 2016). While the use of AvgMonths as an environmental covariate has somewhat enhanced the accuracy of the prediction model compared to single-year monthly data, the inherent tendency of greater randomness in single-temporal remote sensing data persists. Under specific climatic conditions, the dynamics of SOC and STN are influenced by soil characteristics and the type of planted vegetation (Zhong et al., 2018). Remote sensing data, including the monthly vegetation index (NDVI), is temporal and experiences seasonal changes due to the influence of environmental factors like precipitation and temperature variations, as well as soil processes (Maynard and Levi, 2017). There is a significant difference in data across different months, exhibiting clear characteristics of the plant growth season.

**Figure 6:**
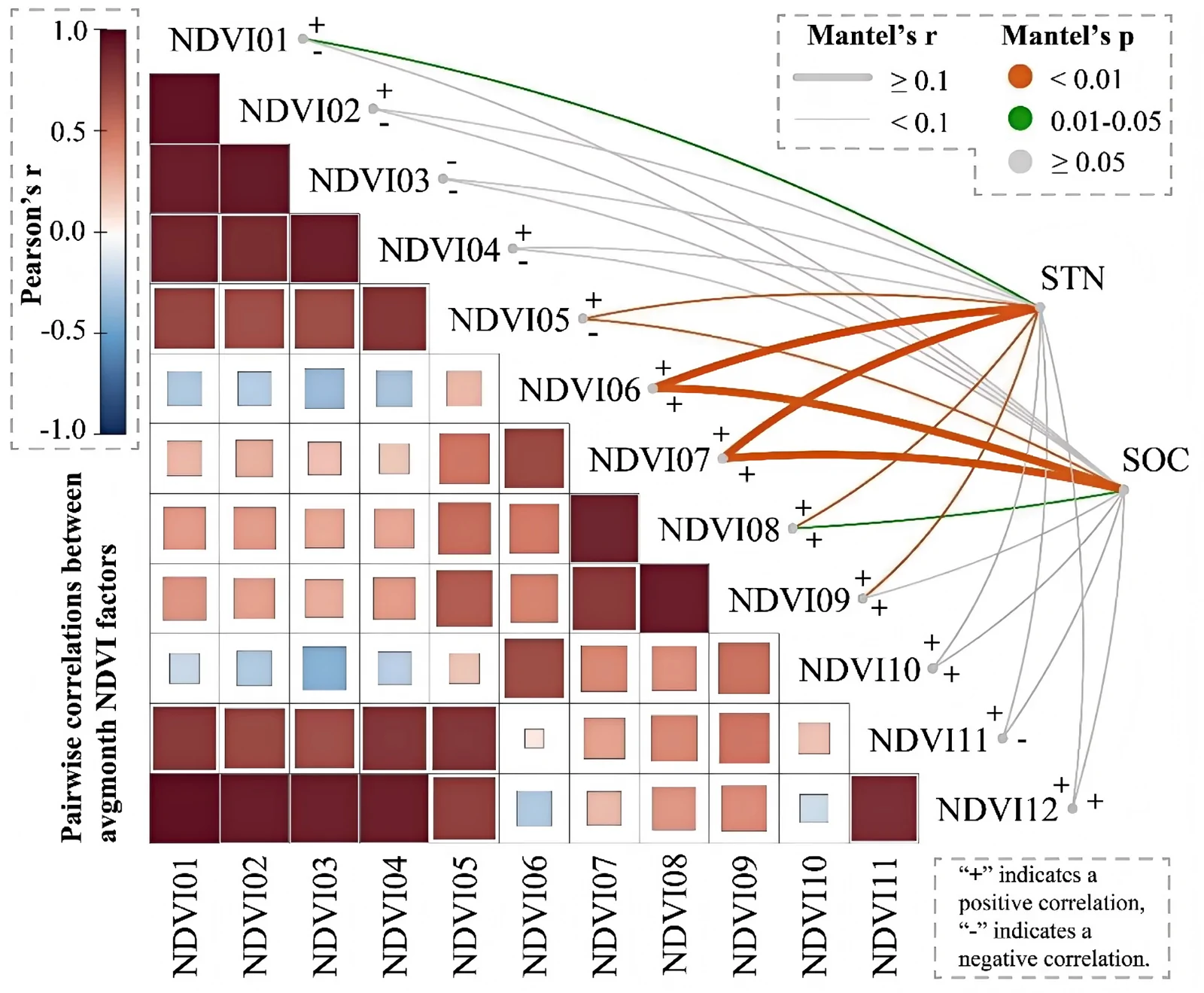
Pearson correlation analysis and conducted Mantel test analysis on the 12-month monthly. average annual data of NDVI for 4 years with respect to STN and SOC.

Meanwhile, as illustrated in **Figure 6**, Pearson correlation analysis of the 12-month monthly average annual data (AvgMonths) of the four-year NDVI and its Mantel test analysis with SOC and STN were conducted. The correlation of NDVI data fluctuates across different months, but the significance between different months is extremely significant with p<0.005. Although the overall distribution of single-temporal NDVI data at the regional scale is similar, the fluctuation of a single month’s data cannot accurately reflect the target soil properties, leading to unstable prediction performance. This aligns with the above analysis results and follows a general pattern. Lower correlations mainly exist between months with a larger span, potentially due to differences in natural factors like seasonality, water temperature, and human factors such as crop vegetation planting affecting NDVI data in different months. The correlation between NDVI data in the single-temporal AvgMonths mode and the target soil attributes is variable, with the correlation coefficient fluctuating. Therefore, when using NDVI data as an environmental covariate in the prediction process of target soil attributes, the prediction performance is unstable when single-temporal NDVI data is employed. Consequently, the use of single-temporal remote sensing data as an environmental covariate often introduces randomness and is susceptible to crop growth cycles, disasters, or human factors. Incorporating multi-temporal remote sensing data into the prediction of SOC and STN allows the observed relationships to be employed in soil-vegetation systems, facilitating the interpretation of soil properties in regions with dense natural vegetation cover (Yang et al., 2019). This approach aids in capturing the comprehensive spatial variations of soil properties and the correlation between NDVI and the target soil properties (Ceddia et al., 2017; Yang and Guo, 2019). Thus, the use of multi-temporal NDVI data and the comprehensive consideration of model effectiveness, simplicity, and efficiency become the focal points of analysis and discussion (Wang et al., 2022).

**Figure 7:**
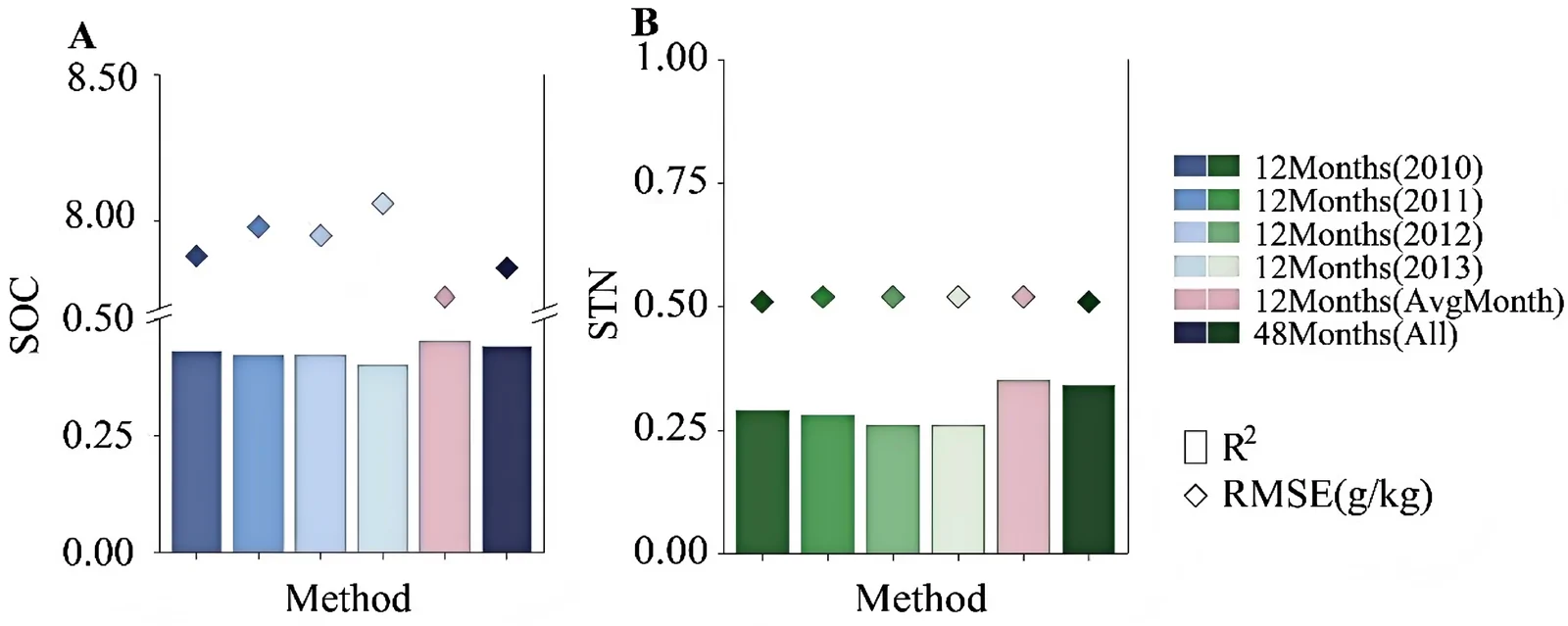
Multi-temporal remote sensing data was used to predict the performance of **(A)** topsoil organic carbon (SOC) and **(B)** topsoil total nitrogen (STN).

Illustrated in **Figures 7A, B**, multi-temporal NDVI data were employed as environmental covariates for predicting SOC and STN in the study area. The prediction model’s R^2^ and RMSE were subsequently determined. Specifically, this involves utilizing all 12 monthly NDVI datasets for each year spanning from 2010 to 2013, monthly averages for the entire year within the same period, and the entirety of the 48 monthly NDVI datasets across the four years from 2010 to 2013 (referred to as All). The results indicate that utilizing multi-temporal NDVI data as environmental covariates significantly enhances forecasting performance in comparison to single- temporal remote sensing data. This improvement is attributed to the ability of multi-temporal remote sensing data to capture vegetation growth cycle changes throughout the year, providing a more comprehensive reflection of soil attribute content (Rizzo et al., 2020). Simultaneously, employing AvgMonths and All yields slightly improved prediction model accuracy compared to using single- year NDVI data as an environmental covariate. This suggests that the growth patterns of vegetation crops in multi-year agricultural areas can be effectively represented by multi-year remote sensing data (Dou et al., 2019). It mitigates the reliance on NDVI data for predicting surface SOC and STN, reducing susceptibility to disasters or human factors in a specific year. In comparison between two methods, AvgMonths and All, the difference in prediction model accuracy is not significant, however, AvgMonths slightly outperforms All. Importantly, AvgMonths not only streamlines the set of covariates, reducing operational complexity, but also incorporates multi-year annual data, diminishing the variability inherent in individual NDVI data. Prior research indicates that judiciously employing remote sensing data fusion enhances the prediction accuracy of DSM for soil properties (Bao et al., 2021; Meng et al., 2022). Hence, considering the study area’s large scale, the model’s effectiveness, simplicity, and operational efficiency are comprehensively taken into account. Multi-year monthly average remote sensing data is utilized as environmental covariates to enhance the prediction of SOC and STN. This approach is more favorable for enhancing model accuracy and accurately depicting the spatial variability of soil attributes. The accuracy presented in Section 3.2 of this study was achieved through the use of the optimal prediction model, wherein the NDVI data from AvgMonths was included in the environmental covariate set for model prediction.

### 4.2 The influence of SOC and STN spatial structural differences and spatial prediction accuracy

**Figure 8:**
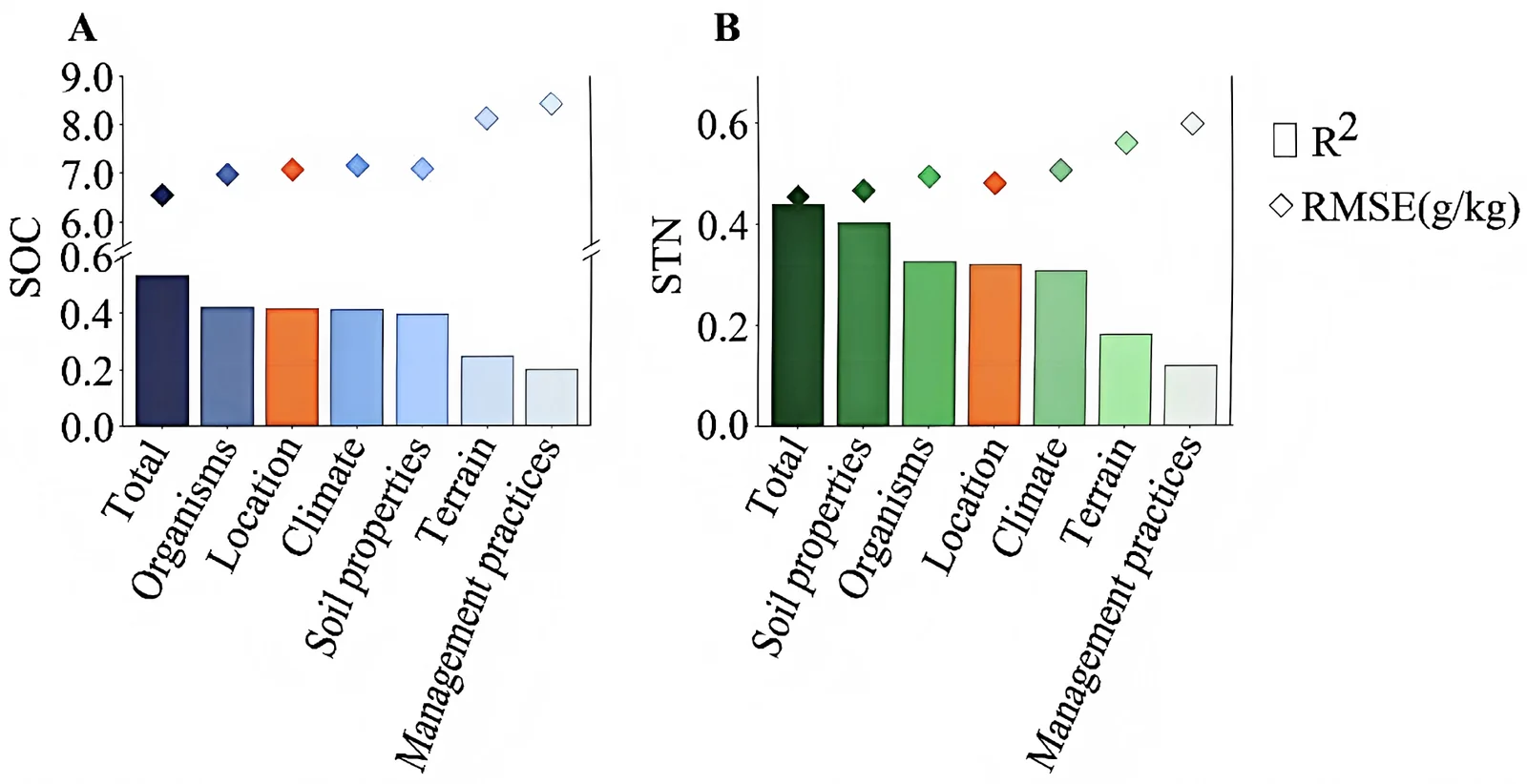
Predictive performance of grouped **(A)** topsoil organic carbon(SOC) and **(B)** topsoil total nitrogen(STN).

The fundamental technical framework for digital soil mapping is the SCORPAN model (soils and/or soil properties, climate and/or climate properties, soils and/or soil Properties, organisms like flora and fauna and human activities, relief settings, parent material, age, and spatial coordinates n) introduced by McBratney et al (McBratney et al., 2003). Exploring the spatial variability of soil attributes is crucial in the construction of the model, especially when considering the large-scale research area. The semi-variance results in Section 3.1 indicate a strong spatial dependence in soil attributes. To explore the spatial structural disparities between SOC and STN, we categorize the environmental covariate data based on spatial coordinates, as detailed in Section 2.5. RF models were constructed separately for each group of environmental covariates, as depicted in **Figure 8**. From a performance perspective, the R² values for the spatial location groups were 0.4 for SOC and 0.3 for STN. The strong predictive power of spatial location can be attributed to the large latitudinal and longitudinal extent of the study area (ca. 31°N–42°N, 110°E–122°E), where spatial coordinates integrate the heterogeneity of climate, soil, and cropping systems, which together shape the regional patterns of carbon and nitrogen dynamics (Zeraatpisheh et al., 2022). Both variables exhibited spatial structure, with SOC showing a stronger spatial structure than STN. On the other hand, STN exhibits weak spatial dependence characteristics, and its spatial variation is significantly influenced by random factors, making spatial prediction more challenging and uncertain (Xia et al., 2022). Combining the analysis of the results in section 3.2, it is observed that projected coordinates y is the most crucial environmental covariate for SOC, suggesting that SOC exhibits specific regular variability characteristics in the north-south direction. Therefore, when applying the RF model for predicting SOC and STN, especially on a regional scale, spatial coordinates representing spatial position information can be included as environmental covariates, enhancing the prediction accuracy of the model (Fan et al., 2023).

### 4.3 Influencing factors of SOC and STN spatial variability

The spatial variability of soil properties is shaped by the interplay of various soil-forming factors, however, collecting all influencing factors can be challenging (Lu et al., 2019). Firstly, the soil-forming factors influencing the spatial variability of soil properties are numerous. Secondly, some influencing factors are challenging to express in a data-based manner. Thirdly, they are influenced by human factors, introducing additional randomness. Fourthly, it is challenging to collect comprehensive data for potential environmental covariates at regional and larger scales. Hence, given the extensive and intricate influencing factors, it is beneficial to investigate more comprehensive, effective, and scientifically sound environmental covariates as input variables in studying the spatial variability of SOC and STN. This aids in enhancing the prediction accuracy of the model. Advocate for a more accurate method of predicting spatial variability. In this study, we utilized the SCORPAN model, commonly employed in SOC and STN prediction studies, as the foundation to refine and broaden the covariate set. This set encompasses spatial location, climate characteristics, topographic attributes, biological characteristics, management measures, soil properties, and more, forming the basis of our investigation. Taking into account Section 4.1 and Section 4.2, the outcome presented in Section 3.3 represents the most accurate method in this study.

As indicated in Section 3.2, environmental covariates influence the spatial variability characteristics of soil properties, as illustrated in **Figure 3**; most of the identified important environmental covariates are natural factors. Organisms data is a confirmed environmental covariate, with the importance of NDVI data varying month by month. Spatial location, especially latitude coordinates, holds the top position in the importance ranking. The research indicates that at the provincial scale, particularly evident in regional and larger scales, the research area exhibits notable differences between the northern and southern regions due to various environmental factors such as light and temperature variations, soil parent material, and soil type. The spatial variability of these soil properties tends to be more pronounced in the north-south direction. In addition to that, cation exchange capacity (CEC) is an index measuring the soil’s ability to retain positively charged ions. Its content impacts the structural stability and nutrient availability of other soil properties (Solly et al., 2020). Clay minerals and organic carbon in soil possess negatively charged sites on their surfaces, enabling the adsorption and retention of positively charged ions through static electricity. Hence, the spatial relationship among soil CEC, clay content, and SOC is not consistent but varies, influencing the spatial variability characteristics of SOC and STN. Besides soil properties, natural environmental factors like precipitation and air temperature exert notable influences on topsoil properties. A prerequisite for the net accumulation of soil organic carbon (like SOC) is that its formation rate outpaces its decomposition rate, a process that is closely regulated by precipitation (Garcia-Palacios et al., 2021; Shen et al., 2023). In arid areas, the low biological density, influenced by soil and water conditions, hampers the generation of organic carbon. Conversely, in humid regions, conducive to organism growth, organic carbon generation is promoted, and air temperature influences its decomposition. Higher temperatures accelerate the production of soil organic carbon, whereas lower temperatures decelerate its decomposition (Wang et al., 2018). Hence, MAT and MAE are the primary factors influencing the spatial variability of soil properties. Previous studies frequently demonstrate the impact of geomorphology and elevation (Gomes et al., 2019; Zeraatpisheh et al., 2019). Elevation is frequently utilized as a pivotal terrain factor in predicting the target soil properties (Taffi, 2019). Variations in elevation can alter heat and water distribution, thereby influencing the availability and spatial variability of soil properties (Martin et al., 2014). Regarding human factors, the study identified straw return amounts, fertilizer application amounts, and LUCC as important covariates in the analysis of SOC spatial variability. However, the ranking of these environmental covariates was not at the top. Additionally, among the confirmed covariates used to predict the spatial variability of STN, LUCC was not included. This indicates that at smaller scales, human factors have a more pronounced relative effect on the spatial differentiation of soil properties. Conversely, at larger scales, natural factors like climate and topography exert a greater relative influence on the spatial differentiation of soil properties.

## 5 Conclusion

In this study, the agricultural area of North China served as the study area, and the random forest model was employed for predicting and mapping SOC and STN. The objective was to explore the spatial variability of these attributes and the variations in their influencing factors across a large region. The main conclusions are as follows: (1) Utilizing spatial location, climate, terrain, organisms, management practices, and soil properties as environmental covariates, the RF model effectively predicts the spatial variability of SOC (R^2^ = 0.53) and STN (R^2^ = 0.44) in agricultural areas of North China. The overall spatial variability characteristics of SOC and STN exhibit similarity, displaying a pattern of “high in the north and low in the south.” In this large-scale spatial variability prediction, key influencing factors include natural environmental factors such as cation exchange capacity (CEC), mean annual temperature (MAT), mean annual elevation (MAE), normalized difference vegetation index (NDVI), spatial coordinates, and elevation. (2) Remote sensing variables play a crucial role as environmental covariates in DSM. Integrating multi-temporal NDVI data into the spatial prediction of SOC and STN reduces prediction performance fluctuations and provides a more accurate reflection of the spatial variability of soil attributes compared to using single-temporal NDVI data. It is advisable to employ the monthly mean annual NDVI data (AvgMonths) as the NDVI data utilization method for predicting SOC and STN. This approach not only enhances model prediction accuracy but also streamlines the covariate set, thereby reducing operational complexity. Additionally, incorporating geographic coordinates as environmental covariates to represent spatial location information improves the prediction accuracy of SOC and STN models. Furthermore, SOC exhibits stronger spatial structure, making spatial location a more beneficial environmental covariate for predicting the spatial variability of SOC.

The spatial prediction variability of SOC and STN in this study is crucial for maintaining the dynamic carbon and nitrogen balance, offering valuable insights for the sustainable development of agriculture and the establishment of ecological civilization in this region. Additionally, it highlights the significance of multi-temporal remote sensing data and spatial location, optimizing the environmental covariate set for DSM. This has considerable implications for the future spatial variability prediction of SOC and STN.

## Data Availability

The raw data supporting the conclusions of this article will be made available by the authors, without undue reservation.
